# Infection of the Asian gray shrew *Crocidura attenuata* (Insectivora: Soricidae) with *Sarcocystis attenuati* n. sp. (Apicomplexa: Sarcocystidae) in China

**DOI:** 10.1186/s13071-021-05136-z

**Published:** 2022-01-11

**Authors:** Junjie Hu, Jun Sun, Yanmei Guo, Hongxia Zeng, Yunzhi Zhang, Jianping Tao

**Affiliations:** 1 School of Ecology and Environmental Sciences and Yunnan Key Laboratory for Plateau Mountain Ecology and Restoration of Degraded Environments, Yunnan University, Kunming, 650091 China; 2 School of Biological Sciences, Yunnan University, Kunming, 650091 China; 3 Haiyuan College, Kunming Medical University, Kunming, 650106 China; 4 Yunnan Institute of Endemic Disease Control and Prevention, Dali, 671000 China; 5 School of Public Health, Dali University, Dali, 671000 China; 6 College of Veterinary Medicine, Yangzhou University, Yangzhou, 225009 China

**Keywords:** *Crocidura attenuata*, *Sarcocystis attenuati*, Life cycle, Morphological and molecular characterization

## Abstract

**Background:**

Data on the genus *Sarcocystis* in insectivores are limited. The Asian gray shrew *Crocidura attenuata* is one of the most common species of the insectivore family Soricidae in South Asia and Southeast Asia. To our knowledge, species of *Sarcocystis* have never been recorded previously in this host.

**Methods:**

Tissues were obtained from 42 Asian gray shrews caught in 2017 and 2018 in China. Sarcocysts were observed using light microscopy (LM) and transmission electron microscopy (TEM). To describe the parasite life cycle, muscle tissues of the host infected with sarcocysts were force-fed to two beauty rat snakes *Elaphe taeniura*. Individual sarcocysts from different Asian gray shrews, and oocysts/sporocysts isolated from the small intestines and feces of the experimental snakes, were selected for DNA extraction, and seven genetic markers, namely, two nuclear loci [18S ribosomal DNA (18S rDNA) and internal transcribed spacer region 1 (ITS1)], three mitochondrial genes [cytochrome oxidase subunit 1 (*cox1*), *cox3* and cytochrome b], and two apicoplast genes (RNA polymerase beta subunit and caseinolytic protease C), were amplified, sequenced and analyzed.

**Results:**

Sarcocysts were found in 17 of the 42 (40.5%) Asian gray shrews. Under LM, the microscopic sarcocysts showed saw- or tooth-like protrusions measuring 3.3–4.5 μm. Ultrastructurally, the sarcocyst wall contained numerous lancet- or leaf-like villous protrusions, similar to those described for type 9h of the common cyst wall classification. The experimental beauty rat snakes shed oocysts/sporocysts measuring 11.9–16.7 × 9.2–10.6 μm with a prepatent period of 10–11 days. Comparison of the newly obtained sequences with those previously deposited in GenBank revealed that those of 18S rDNA and *cox1* were most similar to those of *Sarcocystis scandentiborneensis* recorded in the tree shrews *Tupaia minor* and *Tupaia* *tana* (i.e., 97.6–98.3% and 100% identity, respectively). Phylogenetic analysis based on 18S rDNA or ITS1 sequences placed this parasite close to *Sarcocystis* spp. that utilize small animals as intermediate hosts and snakes as the known or presumed definitive host. On the basis of morphological and molecular characteristics and host specificity, the parasite was proposed as a new species, named *Sarcocystis attenuati*.

**Conclusions:**

Sarcocysts were recorded in Asian gray shrews, to our knowledge for the first time. Based on morphological and molecular characterization, a new species of parasite is proposed: *Sarcocystis* *attenuati*. According to the LM and TEM results, *S. attenuati* sarcocysts are distinct from those of *Sarcocystis* spp. in other insectivores and those of *S*. *scandentiborneensis* in tree shrews. The 18S rDNA or *cox1* sequences of *Sarcocystis attenuati* shared high similarity with those of *Sarcocystis* *scandentiborneensis*, *Sarcocystis zuoi*, *Sarcocystis* cf. *zuoi* in the Malayan field rat, and *Sarcocystis* sp. in the greater white-toothed shrew. Therefore, we suggest that more research on the relationships of these closely related taxa should be undertaken in the future.

**Graphical abstract:**

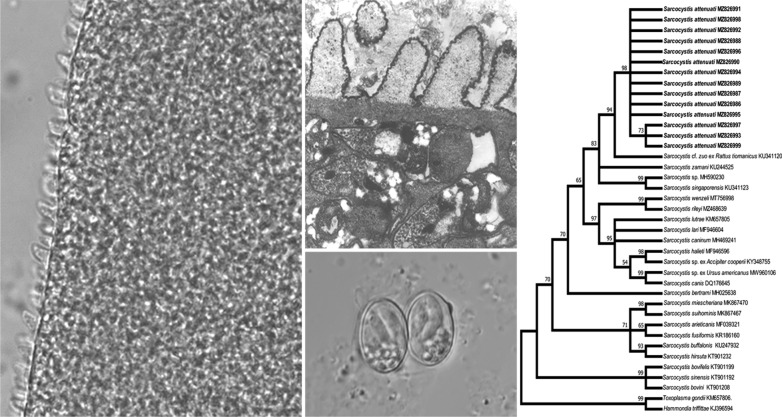

## Background

*Sarcocystis* spp. are cyst-forming intracellular protozoan parasites with an obligate two-host life cycle, with predators as the definitive hosts and their prey as intermediate hosts. More than 200 species of *Sarcocystis* have been described in a variety of wild and domesticated animals [1]. However, only two species, *Sarcocystis* *booliati* and *Sarcocystis russuli*, have been described which are thought to use insectivorous animals as intermediate hosts [1–3]. The order Insectivora comprises approximately 400 species, which makes it the third largest order of mammals after the Rodentia and Chiroptera [4]. The Asian gray shrew *Crocidura attenuata*, a species of the insectivore family Soricidae, is one of the most common species of insectivore found throughout South and Southeast Asia [5]. To our knowledge, no species of the genus *Sarcocystis* have been found and recorded previously in this host.

Here, we describe the morphological and molecular characteristics of a new species of *Sarcocystis* detected in Asian gray shrews. The life cycle of the new species was determined using animal experiments in the laboratory to test a hypothesis arising from phylogenetic analysis, i.e., that snakes probably serve as the definitive hosts of this parasite.

## Methods

### Microscopic examination of sarcocysts from Asian gray shrews

A total of 42 Asian gray shrews were captured from July 2017 to December 2018 by live trapping on farmland in Anning Prefecture, which is located in the mountainous zone of the central part of Yunnan Province, China. All of the captured shrews were euthanized with ether and transported to the zoological laboratory of Yunnan University, Kunming, China, on the same day.

Fresh preparations of the esophagus, diaphragm, tongue, skeletal muscles (thigh, loin, rump, and ribs) and heart of each animal were squeezed between two glass slides and inspected for sarcocysts using a stereomicroscope. Sarcocysts were extracted from muscular fibers using dissection needles and processed for light microscopy (LM), transmission electron microscopy (TEM) and DNA analysis.

For TEM, four sarcocysts obtained from an Asian gray shrew were fixed in 2.5% glutaraldehyde in cacodylate buffer (0.1 M, pH 7.4) at 4 °C, post-fixed in 1% osmium tetroxide in the same buffer, dehydrated in a graded alcohol series, and embedded in Durcupan. Ultrathin sections were double stained with 35 mg/ml uranyl acetate and 35 mg/ml lead citrate and examined using a JEM100-CX transmission electron microscope (JEOL, Tokyo, Japan) at 80 kV.

### Experimental infection of potential definitive hosts

To determine the life cycle of the parasite, three beauty rat snakes *Elaphe taeniura*, purchased from a pet market in Kunming city, were housed separately in steel cages at ambient temperature and humidity. The snakes were force-fed quail eggs, and the feces of the snakes were examined for 2 weeks via a standardized flotation technique using a saturated salt solution (density of 1.20) to confirm that they were free of coccidia.

Pieces of muscle from a wild-caught Asian gray shrew were force-fed to two of the beauty rat snakes, and the third snake was kept as a control. The experimental snakes were each fed 10-g muscle pieces containing approximately 300 sarcocysts (average density 30/g), and fecal samples of the snakes were examined daily for 2 weeks post-infection (PI) via the flotation method to determine the presence of oocysts/sporocysts. All three snakes were euthanized at 29 days PI. The small intestine of each snake was removed and digested (1% trypsin, 37 °C, 3 h), and the digested tissue was filtered and centrifuged (367 *g*/min, 10 min). The sediments were screened for the presence of oocysts/sporocysts using LM. Oocysts/sporocysts from the feces and small intestines of the experimental snakes were collected and stored in deionized water at 4 ℃ for DNA extraction.

### Molecular characterization

A total of 11 samples, comprising ten individual sarcocysts obtained from different Asian gray shrews and oocysts/sporocysts (approximately 250) collected from the experimental snakes, were subjected to DNA extraction using the TIANamp Genomic DNA Kit (Tiangen Biotech, Beijing, China) according to the manufacturer’s instructions. The sarcocysts and oocysts/sporocysts were characterized on the basis of seven genes, namely, two nuclear loci [18S ribosomal DNA (18S rDNA) and internal transcribed spacer region 1 (ITS1)], three mitochondrial genes [cytochrome oxidase subunit 1 (*cox1*), *cox3* and cytochrome b (*cytb*)], and two apicoplast genes [RNA polymerase beta subunit (*rpoB*) and caseinolytic protease C (*clpC*)]. The primers used to amplify these genes are given in Table 1.

**Table 1:** Primers used for the amplification of seven DNA regions

DNA region	Primer name	Primer sequence (5′–3′)	Reference
18S rDNA	ERIB1^a^	ACCTGGTTGATCCTGCCAG	[6]
	S2^b^	CTGATCGTCTTCGAGCCCCTA	[7]
	S3^a^	TTGTTAAAGACGAACTACTGCG	[7]
	B^b^	GATCCTTCTGCAGGTTCACCTAC	[8]
ITS1	ITSF^a^	GTTCCGGTGAATTATTCGGACTGTT	This study
	ITSR^b^	GATGATTCCCTGAATTCTGCAATTC	This study
*cox1*	526F1^a^	TCCTTCCTGGCGTACAACAATCAT	This study
	1209R1^b^	GGGGCATGACATTGAAAGCAAGTA	This study
*cox3*	COX3F1^a^	GCTTTAAACGTATTGTATTTCAATA	This study
	COX3R1^b^	TCAACCATAGACATTCTATGAAATG	This study
*cytb*	1080CYTBF2^a^	ATGAGTTTAGTGCGAGCACATTT	This study
	1080CYTBR2^b^	TTAATATAGACATACAGCTAAGCTTGTGA	This study
*rpoB*	RpoBF^a^	ATTTTTGTGGATATGATTTTGAAGATGC	[9]
	RpoBR^b^	TTTCCATATCTTCCACATAATTTATCTC	[9]
*clpC*	ClpCF-1^a^	GGAGCACCACCTGGGTATGT	This study
	ClpCR-1^b^	CGAGCTCCATATAAAGGATGATAAG	This study

The primers used in this study for internal transcribed spacer region 1 (ITS1), cytochrome oxidase subunit 1 (*cox1*), *cox3*, cytochrome b (*cytb*), and caseinolytic protease C (*clpC*) were designed using OLIGO 5.0 (National BioScience, Plymouth, MN) based on the highly conserved regions of the corresponding sequences for *Sarcocystis* spp., *Toxoplasma gondii*, *Besnoitia besnoiti* and *Hammondia heydorni* deposited in GenBank

18S rDNA 18S ribosomal DNA, *rpoB* RNA polymerase beta subunit

^a^Forward primer

^b^Reverse primer

Polymerase chain reaction (PCR) was performed in a reagent mixture with a total volume of 25 µl that included 12.5 µl Green Taq Mix (Vazyme Biotech, Nanjing, China), 5.5 µl double-distilled H_2_O, 1.0 µl of each primer (10 µM), and 5 µl template DNA. The cycling parameters differed for each gene. For 18S rDNA and ITS1, the amplification reaction started with denaturation at 94 °C for 5 min, followed by 35 cycles of 94 °C for 1 min, 57 °C for 1 min, and 72 °C for 1.5 min, with a final extension at 72 °C for 10 min. For *cox1* and *cox3*, the cycling parameters started with denaturation at 94 °C for 5 min, followed by 35 cycles of 94 °C for 1 min, 54 °C for 1 min, and 72 °C for 1.5 min, with a final extension at 72 °C for 7 min. For *cytb*, *rpoB* and *clpC*, the cycling parameters started with denaturation at 94 °C for 5 min, followed by 35 cycles of 94 °C for 1 min, 52 °C for 1 min, and 72 °C for 1.5 min, with a final extension at 72 °C for 7 min. The PCR products were purified, cloned, sequenced, and assembled using the methods described in our previous paper [10].

Initial screening using the Basic Local Alignment Search Tool showed that the newly obtained 18S rDNA and ITS sequences shared high similarity with those of known definitive host snakes belonging to the genus *Sarcocystis*; no or few nucleotide sequences of the *cox1*, *cytb*, *rpoB*, and *clpC* genes of this taxon have been deposited thus far in GenBank. Therefore, only 18S rDNA and ITS1 sequences of the new species were used to infer relationships with other *Sarcocystis* spp. using MegaX software [11]. At these two loci, there was high similarity between *S*. *zuoi* detected in the Malayan field rat *Rattus tiomanicus* by Watthanakaiwan et al. [12], and the parasite found in the Asian gray shrews in the present study. However, the thin-walled sarcocyst of *S*. *zuoi* described by Watthanakaiwan et al. [12] unambiguously differs from the thick-walled sarcocyst of *S. zuoi* proposed by Hu et al. [13] in the Norway rat *Rattus norvegicus*. Therefore, the *S*. *zuoi* in the Malayan field rat is referred to as *Sarcocystis* cf. *zuoi* in this paper to avoid confusing the two parasites. Maximum likelihood trees for 18S rDNA and ITS1 were created with the Hasegawa-Kishino-Yano and Tamura 3-parameter models, respectively, according to the Find Best DNA/Protein Models program integrated into MEGAX. The reliability of the maximum likelihood phylograms was tested with the bootstrap method using 1000 replications.

The 18S rDNA and ITS1 sequences of *Sarcocystis* spp. from different hosts were downloaded from GenBank and aligned using the ClustalW program implemented in MEGAX. The final alignment of the 18S rDNA sequences consisted of 40 nucleotide sequences and 1668 aligned positions of 35 taxa. *Cystoisospora ohioensis* (GU292304), *Besnoitia besnoiti* (DQ227418), *Hammondia heydorni* (GQ984224), and *Toxoplasma gondii* (U03070) were chosen as outgroups. The final alignment of ITS1 sequences consisted of a total of 39 nucleotide sequences and 1123 aligned positions of 26 taxa. *T. gondii* (KM657806) and *Hammondia* *triffittae* (KJ396594) were used as outgroup species to root the tree.

## Results

### LM and TEM of sarcocysts

**Fig. 1a–e:**
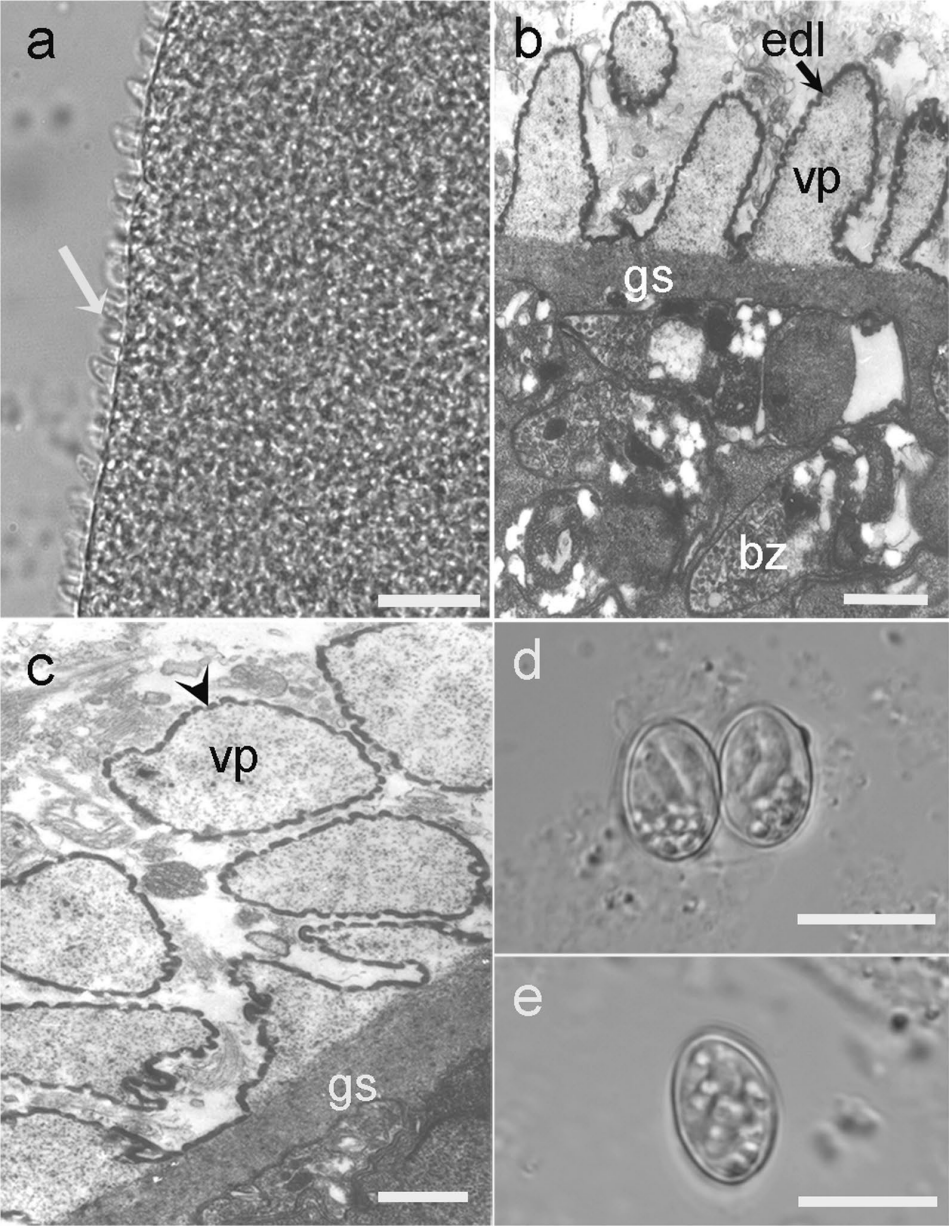
Stages of *Sarcocystis attenuati* n. sp. **a** Light micrograph of a sarcocyst from a fresh sample of skeletal muscle of an infected Asian gray shrew *Crocidura attenuata* (unstained). Note the saw- or tooth-like villous protrusions (*arrow*). *Scale bar* 20 μm. **b** Transmission electron micrograph of the cyst wall of a sarcocyst in longitudinal section. Note the lancet- or leaf-like villous protrusion (*vp*), lined with an electron-dense layer (*edl*). The ground substance (*gs*) is located beneath the vp and bradyzoites (*bz*) within the sarcocyst*. Scale bar* 2 μm. **c** Transmission electron micrograph of the cyst wall of a sarcocyst in cross-section. Note the minute undulations over the surface of the vp (*arrowhead*)*. Scale bar* 2 μm. **d** Light micrograph of sporulated oocysts in the feces of experimentally infected beauty rat snakes *Elaphe taeniura. Scale bar* 10 μm. **e** Light micrograph of a sporocyst in the feces of an experimentally infected beauty rat snake. *Scale bar* 10 μm

Spindle-shaped sarcocysts were found in 17 of the 42 (40.5%) Asian gray shrews and were located in skeletal muscle, the esophagus, the diaphragm, the tongue and the heart. Only one form of sarcocyst was observed. The examination of fresh samples under LM revealed that the cyst walls of the sarcocysts had numerous 3.3- to 4.5-μm-long [mean = 4.0 ± 0.26 μm (± SD);* n* = 25 measurements taken from ten sarcocysts] saw- or tooth-like protrusions (Fig. 1a). Mature sarcocysts were 740–1355 × 117–250 μm [average = 1020 (± 176) × 175 (± 44) μm;* n* = 20 isolated from four Asian gray shrews] in size; they were septate and contained bradyzoites measuring 8.2–10.4 × 2.0–3.0 μm [average = 9.2 (± 0.7) × 2.5 (± 0.3) μm;* n* = 40 measurements taken from two sarcocysts] in size.

Ultrastructurally, the sarcocysts had lancet- or leaf-like villous protrusions measuring 2.8–6.4 μm [mean = 4.5 (± 1.1) μm;* n* = 15], which contained numerous electron-dense granules in their core; microtubules and fibrils were absent. The primary cyst wall had minute undulations over its entire surface, and was lined with an electron-dense layer. The distances between the protrusions varied. A layer of ground substance with a thickness of 1.2–1.6 μm (mean = 1.3 ± 0.1 μm) was located immediately beneath the primary sarcocyst wall. This layer gave rise to thin septa, which divided the cyst interior into a series of compartments. The compartments were filled with numerous bradyzoites and several undifferentiated metrocytes (Fig. 1b, c).

### Infection of the definitive host

The two beauty rat snakes fed muscle tissue from an Asian gray shrew containing sarcocysts excreted sporulated oocysts/sporocysts (Fig. 1d, e) in their feces, one beginning on day 10 PI and the other on day 11 PI. Upon the death of the snakes at day 29 PI, numerous oocysts/sporocysts were also observed in the small intestine. Under LM, the sporulated oocysts measured 11.9–16.7 × 9.2–10.6 μm [average = 13.5 (± 1.3) × 9.9 (± 0.4) μm;* n* = 27], with two elliptical sporocysts measuring 9.2–10.6 × 6.3–6.8 μm [average 9.9 (± 0.4) × 6.6 (± 0.2) μm; (*n* = 30)]. No oocysts/sporocysts were found in the feces or small intestine of the control snake.

### Molecular analysis

Seven genes (18S rDNA, ITS1, *cox1*, *cox3*, *cytb*, *rpoB* and *clpC*) were successfully amplified from ten individual sarcocysts and oocysts/sporocysts. A total of 16 clones, comprising 10 from 10 individual sarcocysts and six from oocysts/sporocysts, were sequenced, assembled, and submitted to GenBank. The ten 18S rDNA sequences obtained from the sarcocysts were 1863–1867 base pairs (bp) in length and shared 99.7–100% identity (average 99.8%). The six 18S rDNA sequences obtained from the oocysts/sporocysts were 1865–1868 bp in length and shared 99.7–100% identity (average 99.8%). Therefore, only three sequences from sarcocysts (accession numbers MZ826981–MZ826983) and two sequences from oocysts/sporocysts (MZ826984 and MZ826985) were deposited in GenBank. The sequence identity between the sarcocysts and oocysts/sporocysts was 99.7–100%, with an average of 99.8%. The most similar sequences in GenBank were those of *Sarcocystis* *scandentiborneensis* (MN733816 and MN733817) obtained from the tree shrews *Tupaia minor* and *Tupaia* *tana* (97.6–98.3% identity, average 97.9%), followed by those of *S. zuoi* (JQ029112 and JQ029113) from the Norway rat *R. norvegicus* (97.1–97.4% identity, average 97.3%), *S.* cf. *zuoi* (KU341118–KU341121) from the Malayan field rat *R. tiomanicus* (96.7–98.0% identity, average 97.2%), *Sarcocystis* sp. (AB251613) from the raccoon *Procyon lotor* (95.9–96.9% identity, average 96.3%), and *Sarcocystis* *clethrionomyelaphis* (KP057504, KF309700, and KF309701) from the large oriental vole *Eothenomys miletus* (95.9–96.4% identity, average 96.2%).

The ten ITS1 sequences obtained from sarcocysts were 874–978 bp in length and shared 97.9–100% identity (average 99.0%). The six ITS1 sequences obtained from oocysts/sporocysts were 875–877 bp in length and shared 98.6–100% identity (average 99.0%). Therefore, only 14 sequences, comprising nine from sarcocysts (MZ826986–MZ826994) and five (MZ826995–MZ826999) from oocysts/sporocysts, were deposited in GenBank. The similarity between the sarcocysts and oocysts/sporocysts was 97.4–100%, with an average of 98.7%. The most similar sequences in GenBank were those of *S*. cf. *zuoi* (KU341118–KU341121) from the Malayan field rat, but the sequence identity was only 72.4–86.0% (average 76.4%).

The ten *cox1* sequences obtained from sarcocysts were 1333 bp in length and shared 99.8–100% identity (average 99.9%). The six *cox1* sequences obtained from oocysts/sporocysts were 1333 bp in length and shared 99.9–100% identity (average 99.9%). Therefore, only five *cox1* sequences, comprising three from sarcocysts (MZ889669–MZ889671) and two from oocysts/sporocysts (MZ889672 and MZ889673), were deposited in GenBank. The similarity between the sarcocysts and the oocysts/sporocysts was 99.8–100%, with an average of 99.9%. The most similar sequences in GenBank were those of *Sarcocystis* *scandentiborneensis* (MN732561 and MN732562, 100% identity), followed by those of *Sarcocystis* sp. (MT411016, 99.8% identity) from the greater white-toothed shrew *Crocidura russula*, and *Sarcocystis* *canis* (KX721496 and KX721497) from the Indo-Pacific bottlenose dolphin *Tursiops aduncus* (95.0–95.4% identity, average 95.2%).

The 16 *cox3* sequences from the sarcocysts and oocysts/sporocysts were 675 bp in length and identical. Therefore, only one sarcocyst sequence (OK001462) and one oocysts/sporocyst sequence (OK001463) were deposited in GenBank. No sequences with significant similarity to these sequences were found in GenBank.

The 16 *cytb* sequences from the sarcocysts and the oocysts/sporocysts were 1080 bp in length and identical. Therefore, only one sarcocyst sequence (OK001464) and one oocysts/sporocysts sequence (OK001465) were deposited in GenBank. The most similar sequences in GenBank were those of *Sarcocystis* *falcatula* (MF034168–MF034187) from the budgerigar *Melopsittacus undulatus*, the identity of which was 96.4%.

The 16 *rpoB* sequences obtained from the sarcocysts and the oocysts/sporocysts were 511 bp in length and shared 100% identity. Therefore, only one sarcocyst sequence (OK001466) and one oocysts/sporocysts sequence (OK001467) were deposited in GenBank. The most similar sequence in GenBank was that of *Sarcocystis* *neurona* (GQ851961) obtained from the southern sea otter *Enhydra lutris nereis*, for which the identity was 91.9%.

The 16 *clpC* sequences from the sarcocysts and the oocysts/sporocysts were 534 bp in length and shared 100% identity. Therefore, only one sarcocyst sequence (OK001468) and one oocysts/sporocysts sequence (OK001469) were deposited in GenBank. The most similar sequences were those of *S*. *falcatula* (KP871717) and *S*. *neurona* (KP871716), for which the identity was 92.5%.

### Phylogenetic analysis

**Fig. 2:**
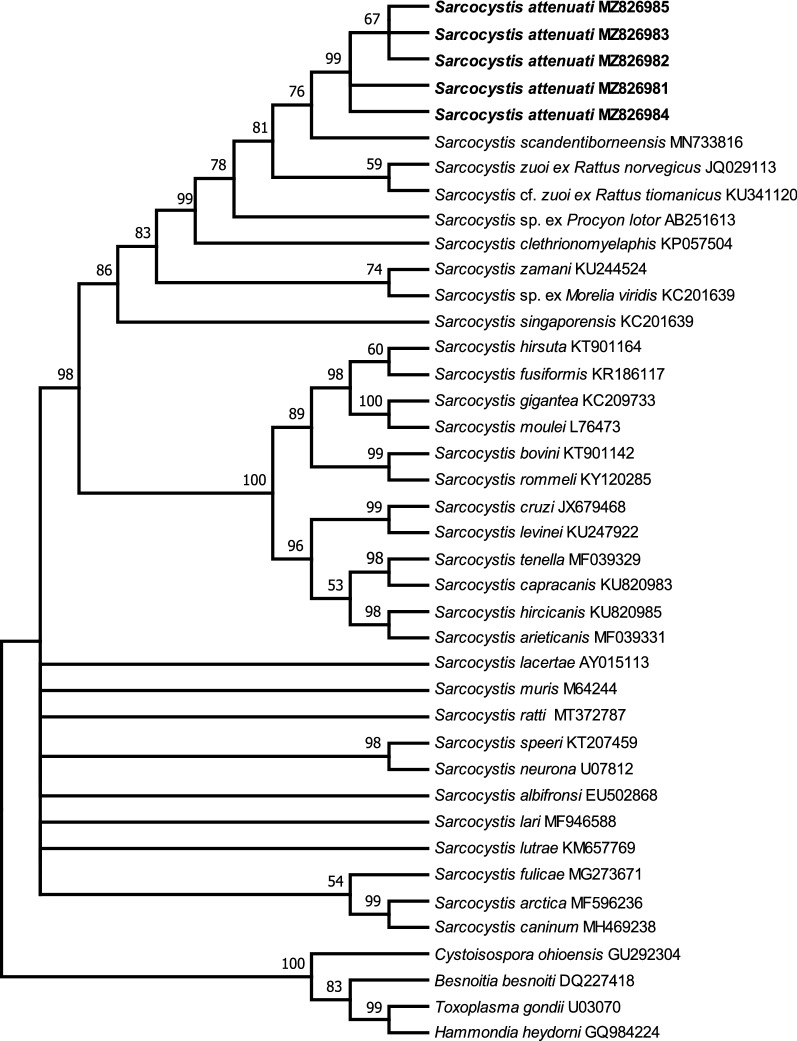
Phylogenetic tree of selected members of the Sarcocystidae based on 18S ribosomal DNA sequences, inferred using the maximum parsimony method with the Tree Bisection and Regrafting algorithm. GenBank accession numbers of all of the sequences included in the analysis are given after the species names. The values between the branches represent percent bootstrap values per 1000 replicates. Bootstrap values below 50% are not shown. The five sequences of *Sarcocystis attenuati* (GenBank MZ826981–MZ826985, shown in* bold*) group within the Sarcocystidae and form a clade with other *Sarcocystis* spp. with snakes as their definitive hosts

**Fig. 3:**
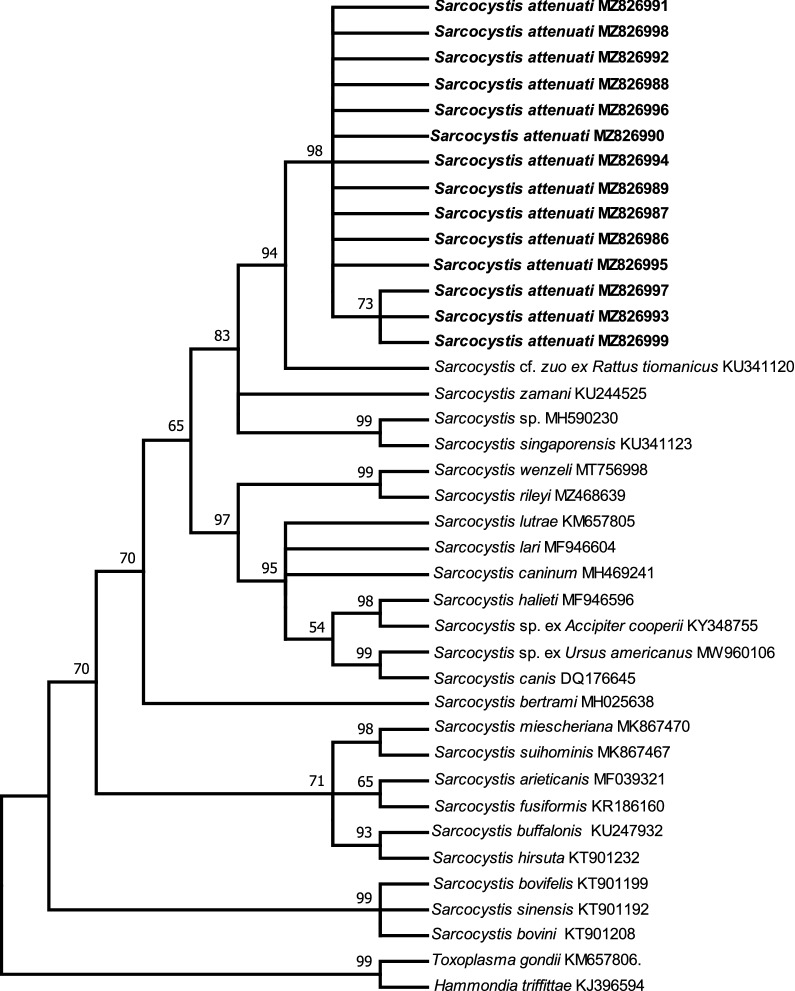
Phylogenetic tree of selected members of the Sarcocystidae based on internal transcribed spacer region 1 sequences, inferred using the maximum parsimony method with the Tree Bisection and Regrafting algorithm. GenBank accession numbers of all sequences included in the analysis are given after the species names. The values between the branches represent percent bootstrap values per 1000 replicates. Bootstrap values below 50% are not shown. The 14 sequences of *Sarcocystis attenuati* (GenBank MZ826986–MZ826999, shown in* bold*) group within the Sarcocystidae and form a clade with other *Sarcocystis* spp. with snakes as their definitive hosts

Phylogenetic analysis based on 18S rDNA or ITS1 sequences confirmed that the parasite found in the present study belonged to *Sarcocystis* (Figs. 2, 3). In the phylogenetic tree inferred from 18S rDNA sequences (Fig. 2), the parasite formed an individual clade and clustered with *Sarcocystis* spp. that use colubrids as their definitive or presumed definitive hosts [i.e., *Sarcocystis* *scandentiborneensis* (MN733816), *Sarcocystis* *zuoi* from *R*. *norvegicus* (JQ029113), *Sarcocystis* cf. *zuoi* from *R*. *tiomanicus* (KU341120), *Sarcocystis* *clethrionomyelaphis* (KP057504), *Sarcocystis* sp. from *Procyon lotor* (AB251613), *Sarcocystis* sp. from *Morelia viridis* (KC201639), *Sarcocystis* *zamani* (KU244524) and *Sarcocystis* *singaporensis* (AF434054)]. In the phylogenetic tree inferred from the ITS1 sequences, the parasite formed an individual group and clustered with *Sarcocystis* spp., with snakes as the definitive or presumed definitive hosts [i.e., *S*. cf. *zuoi* from *R*. *tiomanicus* (KU341120), *S*. *zamani* (KU244525), *Sarcocystis* sp. (MH590230) and *S*. *singaporensis* (KU341123)].

Based on the morphological characteristics of the sarcocysts, molecular analysis and host specificity, the organism found in the Asian gray shrews from Anning Prefecture, China is proposed as a new species with the name *Sarcocystis attenuati*.

### Taxonomic summary of *Sarcocystis attenuati* n. sp.

#### Diagnosis

 The sarcocysts were microscopic, 740–1355 μm long, and 117–250 μm wide. The sarcocysts had 3.3- to 4.5-μm-long saw- or tooth-like protrusions. The sarcocysts were divided by septa into a series of internal compartments filled with bradyzoites measuring 8.2–10.4 × 2.0–3.0 μm. TEM revealed sarcocysts with lancet- or leaf-like protrusions measuring 2.8–7.4 × 0.4–0.9 μm (*n* = 15), which contained electron-dense granules in their core. The protrusions were separated from each other at variable distances, similar to wall type 9h in Dubey et al.’s classification [1].

#### Type (natural) intermediate host

Asian gray shrew *Crocidura attenuata*.

#### Type locality

Anning Prefecture (25°24′N, 102°35′E; altitude 1894 m), China.

#### Site of infection

Muscular tissues throughout the body, including the heart.

#### Experimental definitive host

Beauty rat snake *Elaphe taeniura*.

#### Prevalence

Sarcocysts were found in 17 of the 42 (40.5%) Asian gray shrews examined.

#### Etymology

The species is named after its intermediate host species.

#### Molecular characterization

Nucleotide sequences of the 18S rDNA (MZ826981–MZ826985), ITS1 (MZ826986–MZ826999), *cox1* (MZ889669–MZ889673), *cox3* (OK001462 and OK001463), *cytb* (OK001464 and OK001465), *rpoB* (OK001466 and OK001467), and *clpC* (OK001468 and OK001469) of the new species have been deposited in GenBank. *S*. *attenuati* may be differentiated from *S*. *scandentiborneensis*, which was found in lesser and large tree shrews, according to 18S rDNA sequences. However, the partial *cox1* sequences could not be distinguished from those of *S*. *scandentiborneensis*, or even those of *Sarcocystis* sp. obtained from the greater white-toothed shrew within the covered region of this gene.

#### Deposited specimens

Formalin-fixed tissues containing cysts of *S*. *attenuati*, as well as photomicrographs obtained from the LM and TEM of the sarcocysts, have been deposited at the Zoological Specimen Museum of Yunnan University, Kunming, China (collection no. Pro2018004).

#### ZooBank registration

To comply with the regulations set out in article 8.5 of the amended 2012 version of the International Code of Zoological Nomenclature [14], details of the new species have been submitted to ZooBank. The Life Science Identifier (LSID) of the article is urn:lsid:zoobank.org:pub: 33DEF1BE-BAC4-4E50-8B12-97D9AB1701D8. The LSID for the new species name *Sarcocystis attenuati* n. sp. is urn:lsid:zoobank.org:act: 5CE48112-E64D-496D-8386-4FA2C4F6200A.

### Remarks

The structure of the sarcocyst wall is a useful taxonomic criterion for differentiating *Sarcocystis* spp. within a host. Dubey et al. [1] grouped sarcocysts by sarcocyst wall ultrastructure into 42 wall types with several subgroups. To the best of our knowledge, sarcocysts have been observed in only a few species of insectivores, including the shrew mole *Urotrichus talpoides* in Japan [15], the moonrat *Echinosorex gymnurus* in Malaysia [2], the short-tailed shrew *Blarina brevicauda* in the USA [16], the white-toothed shrew *Crocidura russula* in Russia [3], the common shrew *Sorex araneus*, and the Eurasian pygmy shrew *Sorex* *minutus* in Lithuania [17, 18]. Unfortunately, most of the sarcocysts in these cases were not described in detail. Based on the morphological characterization of their sarcocysts, only two species, *Sarcocystis* *boollati* and *Sarcocystis russuli,* which were found in the moonrat and white-toothed shrew, respectively, have been proposed. Under LM, both species exhibit smooth, thin sarcocyst walls (< 1 μm) [2, 3]. Ultrastructurally, *S*. *booliati* sarcocysts show small knob-like protrusions, similar to wall type 1b [19]. Additionally, Grikieniené [18] found large sarcocysts (up to 5.0–10.0 mm) in the common shrew, which could be seen with the naked eye, and the cyst wall of the sarcocysts was smooth and thin (< 1 μm). Here, sarcocysts of *S*. *attenuati* n. sp. found in Asian gray shrews were microscopic and exhibited a thick cyst wall (3.3–4.5 μm in length), similar to wall type 9h, and they were unambiguously different from those of *S*. *boollati*, *S. russuli* and *Sarcocystis* sp. obtained from the moonrat, white-toothed shrews, and the common shrew, respectively*.*

## Discussion

Insectivores are an abundant group of mostly small mammals, including hedgehogs, moonrats, shrews, and moles. In the present study, an examination of sarcocysts in muscle samples from Asian gray shrews revealed that the prevalence of infection was 40.5% (17/42), which was higher than the 28.6% (2/7) prevalence reported in shrew moles in Japan [15], 1.2% (9/233) prevalence in common shrews and 4.3% (1/23) prevalence in Eurasian pygmy shrews from Lithuania [18]. This high infection rate is probably related to the high abundance of the definitive host in the same area. The prevalence of *S*. *clethrionomyelaphis* in large oriental voles captured in the same area between 2012 and 2014 was as high as 75.8% (47/62) [20]. In both the present study and a previous study [20], we have proven, via transmission experiments, that *S*. *attenuati* and *S*. *clethrionomyelaphis* use the same definitive host, the beauty rat snake.

Nucleotide sequence analysis has been suggested to be a more useful tool for the delineation or identification of *Sarcocystis* from the same host or different hosts. Additionally, different genetic markers have revealed different levels of intra- or interspecific sequence diversity [21–23]. To date, only a few *cox1* sequences of *Sarcocystis* obtained from insectivores, i.e., *Sarcocystis* spp. obtained from the greater white-toothed shrew, have been deposited in GenBank. The similarity between the *cox1* sequences of *S*. *attenuati* from the present study and *Sarcocystis* sp. (MT411016) from the heart of a greater white-toothed shrew from Spain was as high as 99.8%, which suggests that the latter may be *S*. *attenuata*. However, only PCR and Sanger sequencing methods have been used to detect *Sarcocystis* in the greater white-toothed shrew, and sarcocysts of the species have not been described [24]. Interestingly, the 18S rDNA and *cox1* sequences of *S. attenuati* shared high similarity in the regions covered with those of *S*. *scandentiborneensis* (i.e., 97.6–98.3% and 100%, respectively). *S. scandentiborneensis* was found in lesser and large tree shrews, which belong to the order Scandentia, collected from Malaysia. Under LM, the cyst wall of *S. scandentiborneensis* presented tightly packed finger-like protrusions (2–10 μm in length) that can assume a brush-like appearance; ultrastructurally, these protrusions contain bundled microtubules that extend into the ground substance [25]; these characteristics are similar to those of wall type 11b or 12. Thus, based on differences in their morphological characteristics and host specificity, it is proposed that *S. attenuati* is distinct from *S. scandentiborneensis*.

The phylogenetic relationships among the majority of analyzed *Sarcocystis* spp. suggest their coevolution with their definitive hosts rather than with their intermediate hosts [26]. The phylogenetic trees based on 18S rDNA sequences and ITS1 sequences revealed that *S*. *attenuati* formed an individual clade with *Sarcocystis* spp. from small mammals which use snakes as their definitive or putative definitive hosts. The infection experiment confirmed the hypothesis arising from the phylogenetic analysis, and proved that beauty rat snakes can serve as an experimental definitive host of *S*. *attenuati.* The beauty rat snake is native to eastern and southeastern Asia and feeds mainly on shrews and rodents, although the consumption of amphibians, reptiles and birds by this species has also been reported [27]. To date, only two species of *Sarcocystis*, *S*. *clethrionomyelaphis* from the large oriental vole and *S*. *zuoi* from the Norway rat, have been proven via transmission experiments to use species of *Elaphe* as definitive hosts [20, 28]. Morphologically, the sarcocysts of *S*. *clethrionomyelaphis* present thin highly folded protrusions, which often bend along the cyst surface [20], similar to wall type 10f; the sarcocysts of *S*. *zuoi* exhibit sloping finger-like protrusions, and the base of the protrusions is highly branched [13, 28], similar to wall type 17. Therefore, the sarcocysts of *S. attenuati* can be easily morphologically differentiated from those of *S*. *clethrionomyelaphis* and *S*. *zuoi*. It is very common for a predator to act as the definitive host of more than one species of *Sarcocystis*. For example, *Python reticulatus* is the definitive host of three species of *Sarcocystis* found in rats (i.e., *Sarcocystis* *singaporensis*, *Sarcocystis* *villivillosi*, and *Sarcocystis* *zamani*) [29].

## Conclusions

In summary, this report demonstrates, to our knowledge for the first time, the presence of microscopic cysts of a species of *Sarcocystis* in Asian gray shrews. From the morphological and molecular characterization of the sarcocysts, we propose a new species of *Sarcocystis* with the species name *Sarcocystis* *attenuati*, after the host in which it was found. Experimental infection revealed that the beauty rat snake can serve as an experimental definitive host of *S*. *attenuati*; this was also supported by the high similarities between the nucleotide sequences of different genes of the sarcocysts and the oocysts/sporocysts of *S*. *attenuati*. The 18S rDNA sequences and *cox1* sequences of *S*. *attenuati* shared high similarities with those of *S*. *scandentiborneensis* from tree shrews. However, the sarcocysts of these two species present different morphological characteristics under LM and TEM. Our understanding of the biodiversity, host specificity and evolution of *Sarcocystis* should improve with the accurate morphological and molecular characterization of more species of this genus from different insectivores and other small mammals.

## Data Availability

The datasets used and/or analyzed during the current study are available from the corresponding author upon reasonable request. Nucleotide sequences of the 18S rDNA (MZ826981–MZ826985), ITS1 (MZ826986–MZ826999), *cox1* (MZ889669–MZ889673), *cox3* (OK001462 and OK001463), *cytb* (OK001464 and OK001465), *rpoB* (OK001466 and OK001467), and *clpC* (OK001468 and OK001469) of the new species have been deposited in GenBank.

## Abbreviations

*clpC*: Caseinolytic protease C
*cox1*: Cytochrome oxidase subunit 1
*cytb*: Cytochrome b
ITS1: Internal transcribed spacer region 1
LM: Light microscopy
PCR: Polymerase chain reaction
*rpoB*: RNA polymerase beta subunit
18S rDNA: 18S ribosomal DNA
TEM: Transmission electron microscopy
